# Assessing the spatial heterogeneity of tuberculosis in a population with internal migration in China: a retrospective population-based study

**DOI:** 10.3389/fpubh.2023.1155146

**Published:** 2023-05-30

**Authors:** Honghua Lin, Rui Zhang, Zheyuan Wu, Minjuan Li, Jiamei Wu, Xin Shen, Chongguang Yang

**Affiliations:** ^1^School of Public Health (Shenzhen), Shenzhen Campus of Sun Yat-sen University, Shenzhen, China; ^2^Division of TB and HIV/AIDS Prevention, Shanghai Municipal Center for Disease Control and Prevention, Shanghai, China; ^3^Shanghai Institutes of Preventive Medicine, Shanghai, China; ^4^School of Public Health, Yale University, New Haven, CT, United States; ^5^Nanshan District Center for Disease Control and Prevention, Shenzhen, Guangdong Province, China

**Keywords:** tuberculosis, internal migrants, spatial heterogeneity, Bayesian disease mapping, cluster

## Abstract

**Background:**

Internal migrants pose a critical threat to eliminating Tuberculosis (TB) in many high-burden countries. Understanding the influential pattern of the internal migrant population in the incidence of tuberculosis is crucial for controlling and preventing the disease. We used epidemiological and spatial data to analyze the spatial distribution of tuberculosis and identify potential risk factors for spatial heterogeneity.

**Methods:**

We conducted a population-based, retrospective study and identified all incident bacterially-positive TB cases between January 1st, 2009, and December 31st, 2016, in Shanghai, China. We used Getis-Ord Gi* statistics and spatial relative risk methods to explore spatial heterogeneity and identify regions with spatial clusters of TB cases, and then used logistic regression method to estimate individual-level risk factors for notified migrant TB and spatial clusters. A hierarchical Bayesian spatial model was used to identify the attributable location-specific factors.

**Results:**

Overall, 27,383 bacterially-positive tuberculosis patients were notified for analysis, with 42.54% (11,649) of them being migrants. The age-adjusted notification rate of TB among migrants was much higher than among residents. Migrants (aOR, 1.85; 95%CI, 1.65-2.08) and active screening (aOR, 3.13; 95%CI, 2.60-3.77) contributed significantly to the formation of TB high-spatial clusters. With the hierarchical Bayesian modeling, the presence of industrial parks (RR, 1.420; 95%CI, 1.023-1.974) and migrants (RR, 1.121; 95%CI, 1.007-1.247) were the risk factors for increased TB disease at the county level.

**Conclusion:**

We identified a significant spatial heterogeneity of tuberculosis in Shanghai, one of the typical megacities with massive migration. Internal migrants play an essential role in the disease burden and the spatial heterogeneity of TB in urban settings. Optimized disease control and prevention strategies, including targeted interventions based on the current epidemiological heterogeneity, warrant further evaluation to fuel the TB eradication process in urban China.

## Introduction

The cumulative reduction in tuberculosis (TB) incidence was still relatively low and fell short of the WHO target. China has the third highest burden of TB disease in the world, with an estimated 784,400 new TB cases in 2021 (1). Furthermore, population migration (2), multidrug resistance (3, 4), and HIV infection (5) contributed substantially to tuberculosis disease both in China and worldwide. Shanghai was one of the megacities with a massive internal migrant population in China, with an estimated 10.47 million internal migrants by the end of 2020 (6). Accordingly, revealing the influential pattern of the internal migrant population in the incidence of TB could help provide better guidance on controlling and preventing the disease.

A combination of hot spot analysis, spatial relative risk, individual factors, and spatial elements analysis may provide pivotal information for evaluating the spatial distribution heterogeneity of infectious diseases and their risk factors. Recently, many studies reported the spatial heterogeneity of infectious diseases with spatial hot spot analysis (7–9). Such analysis also helped to detect the high-risk regions of TB (10, 11). Researchers regarded the high-risk regions as the disease control targets gaining a good control effect (12). In exploring risk factors, the combined analysis of individual and spatial factors may help clarify the causes of the formed TB spatial heterogeneity more comprehensively, which is more conducive to exploring and identifying high-risk regions for possible targeted interventions (13–15).

In the past two decades, China’s leading urban and east coastal cities have experienced massive internal migration from the western and rural regions, where more than 80% of incident TB cases are located. The migrant population had been reported to be associated with increased TB cases in those cities and likely transmitted TB among local residents (16). Although some studies showed that internal migrants were the main driving force of TB (17, 18), few studies provided direct evidence of the role of internal migrants on TB at the county level of the city. Here, we conducted a population-based retrospective study in Shanghai, China, using epidemiological, spatial, and hierarchical Bayesian analysis methods to analyze the spatial heterogeneity of TB patients in the urban region of China and determine the spatial heterogeneity of TB disease. It provided a basis for public health decision-makers to develop targeted prevention and control strategies for TB.

## Materials and methods

### Study setting and design

Shanghai (Supplementary Figure S1) is one of the first-tier cities in China, with internal migrants accounting for 42% of the population (6). According to China’s household registration system, people without Shanghai household registration are regarded as internal migrants and come from the mainland in China (the distribution of population within Shanghai is shown in Supplementary Figure S2A). We conducted a population-based retrospective study on all bacterially-positive TB cases in Shanghai between January 1st, 2009, and December 31st, 2016. In 2005, Shanghai Municipal Center for Disease Control and Prevention started a TB surveillance system. We collected and abstracted TB patients’ social demographic data (sex, age, occupation), epidemiological information (treatment history, treatment outcomes, patient notification source, diagnosis delay, and addresses), and laboratory results (sputum smear test) from the system (Table 1). We analyzed all bacterially-positive TB patients who were notified and who lived in Shanghai municipal city, which was defined as TB patients who had a positive sputum smear-or culture-positive bacterial culture.

**Table 1:** Demographic and clinical characteristics of internal migrant and resident tb cases in Shanghai, 2009–2016.

Characteristics	Migrant patients		Resident patients		*p* value
	*n* = 11,649	(%)	*n* = 15,734	(%)
Gender					<0.001
Female	4,129	(35.45)	3,847	(24.45)	
Male	7,520	(64.55)	11,887	(75.55)	
Age, median years (IQR)	29	(23–41)	57	(43–71)	<0.001^a^
Age group, years					<0.001
0–14	43	(0.37)	40	(0.25)	
15–24	3,499	(30.04)	1,294	(8.22)	
25–34	3,780	(32.45)	1,570	(9.98)	
35–44	1,988	(17.07)	1,227	(7.80)	
45–54	1,138	(9.77)	2,777	(17.65)	
55–64	709	(6.09)	3,474	(22.08)	
≥65	492	(4.22)	5,352	(34.02)	
Occupation					<0.001
Labour worker	4,194	(36.00)	1,466	(9.32)	
Farmer	370	(3.18)	1,302	(8.28)	
Commercial service	686	(5.89)	499	(3.17)	
Medical staff	44	(0.38)	81	(0.52)	
Student/teacher	445	(3.82)	616	(3.92)	
Retirement	459	(3.94)	5,867	(37.29)	
Unemployed	2,256	(19.37)	1,969	(12.51)	
Other	3,195	(27.43)	3,934	(25.00)	
TB history					<0.001
New case	10,546	(90.53)	13,087	(83.18)	
Retreated case	1,103	(9.47)	2,647	(16.82)	
Treatment outcome					<0.001
Cured/completion	10,174	(87.34)	13,006	(82.66)	
Other	1,475	(12.66)	2,728	(17.34)	
Sputum AFB test					<0.001
Positive	8,482	(72.81)	11,894	(75.59)	
Negative	3,136	(26.92)	3,762	(23.91)	
Other	31	(0.27)	78	(0.50)	
Patient source					<0.001
Active screening^b^	476	(4.09)	216	(1.37)	
Passive screening	11,157	(95.78)	15,499	(98.51)	
Other	16	(0.14)	19	(0.12)	
Diagnosis delay					<0.001
0–2 weeks (w)	3,142	(26.97)	3,786	(24.06)	
2 w–1month (m)	3,322	(28.52)	4,876	(30.99)	
1–3 m	4,232	(36.33)	5,856	(37.22)	
3–6 m	692	(5.94)	912	(5.80)	
6 m–1 year	261	(2.24)	304	(1.93)	

IQR, interquartile range; TB, tuberculosis; AFB, acid fast bacilli.

a Result of Wilcoxon rank-sum test.

b Include contactor investigation (10 cases in migrants, 6 cases in residents).

### Data source and definitions

We assigned each patient to a single county based on their address recorded in the routine TB surveillance system at the time of diagnosis. We geocoded addresses using the Google Map tool (R package “ggmap”) and Baidu Map for those address names that cannot be recognized by Google Map (geocoding results were adjusted between the two methods). We also manually checked and geocoded those addresses hard to perform batch geocoding. The region-level spatial data included all the counties according to the national standards. This study excluded the cases located in Chongming Island and cases lacking county-level address information (Supplementary Figure S1A).

We divided the TB patient sources into three categories: active screening (including contact tracing investigation and health examination), and passive screening (including symptom-based visits and referral by community healthcare centers or non-TB-designated facilities). The diagnosis delay time was calculated as the period from the onset of the first symptom(s) possibly related to the TB to the date when the patient first being diagnosed with pulmonary TB.

### Spatial distribution analysis

#### Overview of the spatial distribution of TB

The distribution of the residential address of each TB patient was geocoded and displayed by ArcGIS 10.8 (ESRI Inc., Redlands, CA, United States). We mapped the spatial distribution of internal migrants among all populations based on the 2010 national census data and depicted the overall/internal migrant TB notified incidence rate in Shanghai at the county level in ArcGIS. We also used Inverse Distance Weighting (IDW) in the R package “gstat” to smooth the maps described above.

#### Hot spot analysis of notified TB patients

We used Getis-Ord General *G* and hot spot analysis (Getis-Ord Gi* statistics) from the ArcGIS cluster distribution mapping module to explore the statistically significant high clustering counties of overall TB, internal migrant TB, and resident TB. Briefly, the Getis-Ord Gi* was estimated by comparing the local sum for a feature and its neighbours with the sum of all features (19). We utilized Queen’s case as the spatial relationship conceptualization parameter, which could reduce the effect of irregular area size and shape (20) and was effective in simulating the infectious process of infectious diseases such as TB. We employed false discovery rate (FDR) correction to overcome multiple testing and spatial dependence limitations, which was better than assuming that each local test was performed independently or applying traditional overly conservative multiple testing methods (21). The estimated Z-score and *p* value were used to verify significant hot spots (high clusters) and cold spots (low clusters). The detected statistically significant hot spots mean counties with a high notification rate of TB and are surrounded by other counties with high values as well. The threshold for determining hot spots is *Z*-score > 1.65 and *p* < 0.10.

### Spatial relative risk estimation

The spatial relative risk function in the R package “sparr” (22) was used to compare the kernel density estimation (KDE) of internal migrant TB $\left(\hat{f}\right)$ and resident TB $\left(\hat{g}\right).\hat{r}=\hat{f}/\hat{g}$ presented the spatial relative risk, which was performed by taking the logarithm to treat two layers of observation symmetric. The spatial relative risk was given as

$${\hat{\rho }}_{h}\left(x|X,Y\right)=\log\left[{\hat{f}}_{h}\left(x|X\right)\right]-\log\left[{\hat{g}}_{h}\left(x|Y\right)\right];x\in W$$

where $X=\left\{x_{1},x_{2},\ldots ,x_{n}\right\}$ was the observed points of the internal migrant TB cases, $Y=\left\{y_{1},y_{2},\ldots ,y_{n}\right\}$ was the observed points of the resident TB cases. W was the study region. We applied the adaptive bandwidth into KDE for the reason of the distance between each location varied widely. *h* was the adaptive bandwidth with a symmetric estimation based on a pooled-data pilot density performed by bootstrap (23). The *p* value surfaces were formed via Monte-Carlo (MC) simulation of kernel-estimated risk functions. $\hat{\rho }>0$ indicated a higher localized density of the internal migrant TB relative to the resident TB in the affected spatial areas.

### Hierarchical Bayesian modelling on spatial risk factor analysis

To better understand the distribution and variation of TB patients among the general population, we used a hierarchical Bayesian disease mapping technique to model total TB case counts at the county level as a function of county-level risk factors. We also used the model to identify residual county-level hot spots representing greater than expected TB incidence among the general population, possibly indicating areas with increased disease transmission. The model was given as

$$Y_{i}|E_{i},\lambda_{i}\sim \mathrm{Poisson}\left(E_{i}\lambda_{i}\right);i=1,\ldots ,m;\mathrm{and}$$

$$\ln\left(\lambda_{i}\right)=O_{i}+z_{i}^{T}\gamma +\phi_{i}+\theta_{i}$$

where $Y_{i}$ was the total number of TB cases in county *i*; $E_{i}$ was the expected number of TB cases; $\lambda_{i}$ was the relative risk in county *i*; $O_{i}$ was an offset term representing the log of the total population; and $z_{i}$ was the vector of county-level predictors of TB risk with γ the corresponding vector of regression parameters.

We selected county-specific covariates for inclusion in the model based on previously reported associations with TB disease (24). These covariates included the fraction of the population that has migrated, population density, household size, rooms per household, *per capita* GDP and a binary indicator of whether the county is an industrial hub.

We used the Besag-York-Mollié (BYM) model (25) to account for spatial correlation in the data. Specifically, the $\phi_{i}$ random effects were modelled using the intrinsic conditional autoregressive model while $\theta_{i}$ were modelled as independent and identically distributed Gaussian distributions with zero mean and unknown variance. Again, we opt for weakly informative prior distributions for the random effect variance parameters.

### Statistical analysis

The *chi-square* test or *Fisher* exact test was used to test the significance of differences between groups. Non-normally distributed quantitative variables were expressed as the median and interquartile range (IQR) and tested using the Wilcoxon rank-sum test. Univariable and multivariable logistic regression analyses were used to calculate the adjusted odds ratio (aOR) and 95% confidence interval (95%CI) for risk factors associated with the high clusters. The multivariable logistic regression model used the backward method for independent variable selection. Statistical analyses were performed in R (version 4.2.1).

## Results

### Demographic and clinical characteristics of migrant and resident TB patients

**Figure 1:**
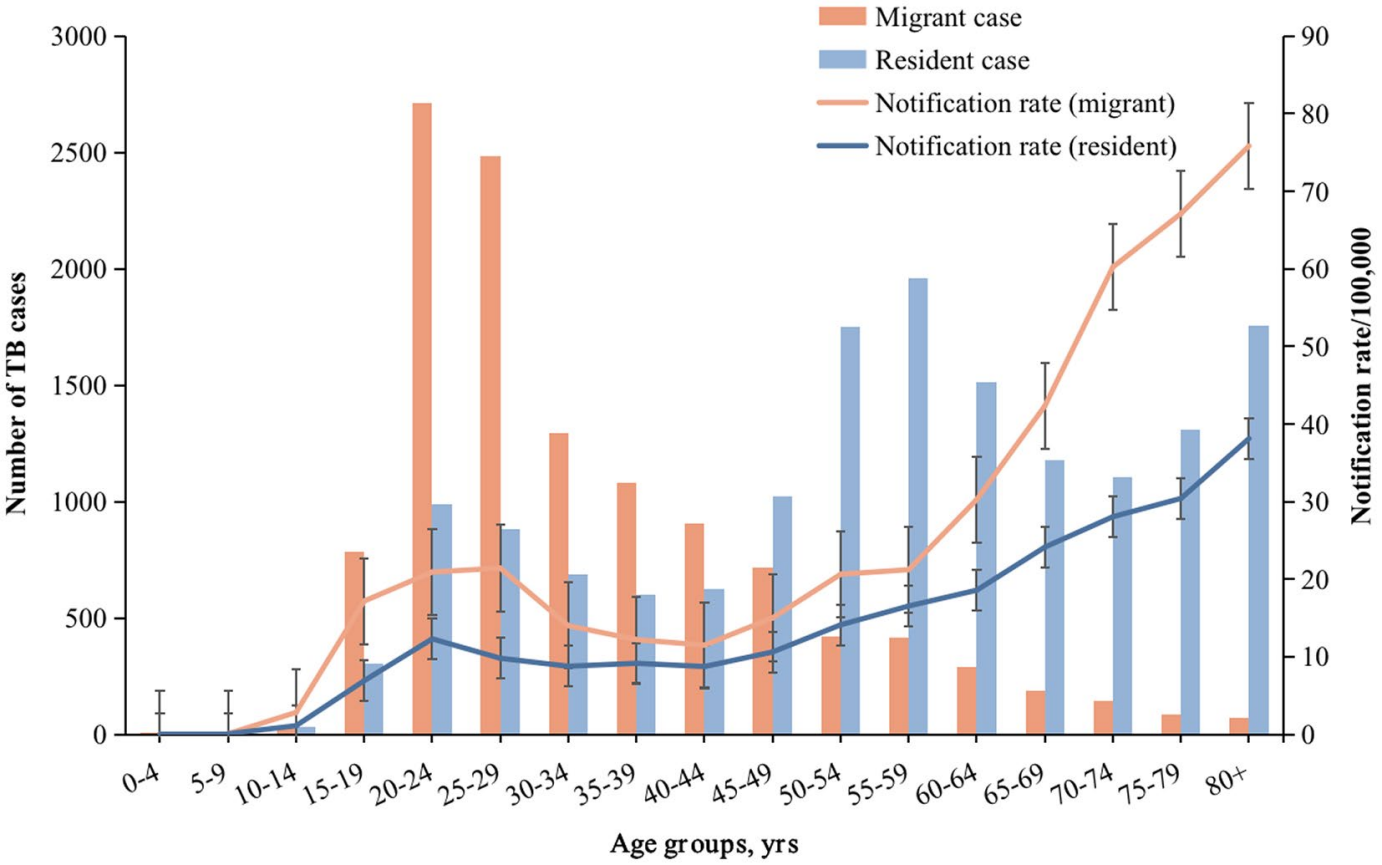
Number of TB cases, notification rate in different age groups of internal migrants and residents. The bar graph represents the number of cases, and the line graph represents the notification rate. Orange and blue correspond to migrant TB and resident TB, respectively.

During the study period, of 61,200 TB cases notified in Shanghai, China, a total of 27,383 bacterially*-*positive TB cases were included in our final analysis (Supplementary Figure S1A). 42.54% (11,649/27,383) of them were internal migrant TB patients. The internal migrant and resident TB were mostly males (64.55% and 75.55%, respectively). The notification rate among migrants was higher than that among residents yearly (Supplementary Figure S3). After age-adjusted estimation, the average standardized annual notification rate of internal migrants (20.98/100,000) was higher than that among residents (12.31/100,000). Figure 1 shows the difference in the standardized rate between these two populations by different age groups.

Table 1 shows the demographic and clinical characteristics of resident and internal migrant TB patients. Internal migrant patients were younger (median age: 29 years vs. 57 years; Table 1; Figure 1; Supplementary Figure S4) and were more likely to be labour workers (36.00% vs. 9.32%) than the resident patients. Internal migrant TB patients were more likely to be notified from active screening than residents (4.09% vs. 1.37%). Both internal migrant and resident patients had a high proportion of diagnosis delay (73.03% and 75.94% with more than 2 weeks delay, respectively), indicating the need for active case finding among both populations.

### Spatial heterogeneity of TB in Shanghai

**Figure 2:**
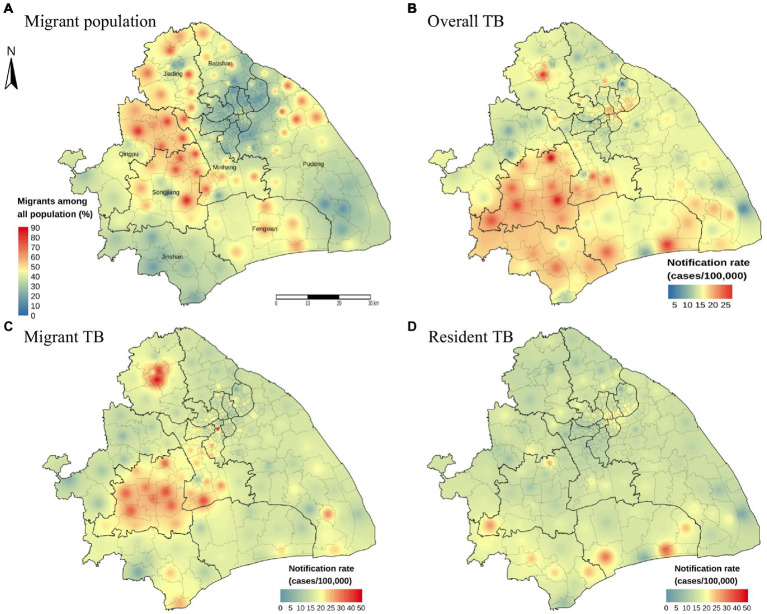
Distribution map of migrant population proportion and notification rate with inverse distance weighted (IDW) smoothing. The proportion of internal migrants among all population **(A)**. The geographic distribution of the notification rate of overall TB **(B)**, internal migrant TB **(C)** and resident TB **(D)** by county level in Shanghai, 2009–2016.

To investigate the role of internal migrants in the burden of TB in the megacity, we first examined the spatial distributions of the average annual notification rate of TB and the proportion of internal migrants by using the IDW method (Figure 2). The average annual notification rate of TB at the county levels ranged from 2.21 to 29.83 cases per 100,000 population among 196 counties in Shanghai. The areas with a high notification rate of TB were mainly in the Songjiang and Minhang Districts, which also had a high proportion of internal migrants among the total inhabitants (Figures 2A,B; Supplementary Figures S2A,B). Overall, the counties with high TB notification rates concordant with those with a high proportion of internal migrants. The central county (Jiading town) of Jiading District had a low proportion of migrants. It had a high TB notification rate, which was more significant for internal migrant TB (Figure 2C). The spatial distribution heterogeneity between internal migrant TB and resident TB was substantial (Figures 2C,D).

**Figure 3:**
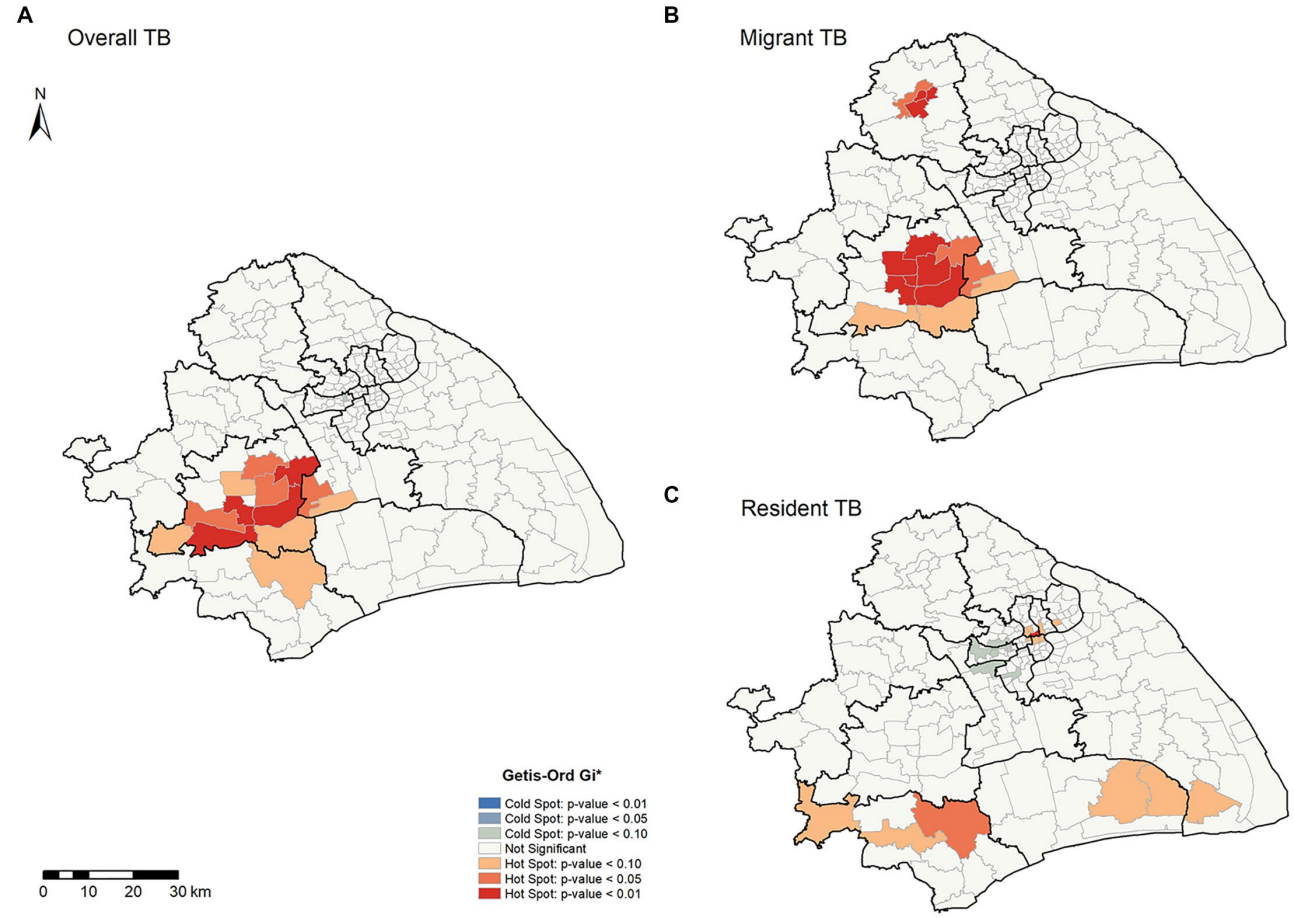
Spatial clustering patterns of TB by county level in Shanghai, 2009–2016. **(A,B,C)** Illustrate the overall, migrant, and resident TB, respectively.

To further explore the spatial heterogeneity of TB in this megacity, we conducted a hot spot analysis using the Getis-Ord General *G* and Getis-Ord Gi* statistics. The result of Getis-Ord General *G* showed that the average annual notification rates of overall TB, internal migrant TB, and resident TB at the county levels all had high-value clustering (*Z*-score > 1.96 and *p* < 0.05) (Supplementary Table S1). Our analysis detected 13 high-clustering counties of overall TB, most of which were in Songjiang and Minhang Districts (Figure 3A). Among migrant TB cases, 15 high-clustering counties were identified, and all were in Songjiang, Minhang, and Jiading Districts (Figure 3B). In Songjiang and Minhang Districts, the high-clustering counties of overall TB were highly consistent with the high-clustering counties of internal migrant TB (the overlapping rate: 76.92%, Supplementary Figure S5). In contrast, the high clusters of resident TB were mainly in Jinshan District (Figure 3C). We observed significant heterogeneity in the distribution of high-clustering counties between internal migrants and residents.

**Figure 4:**
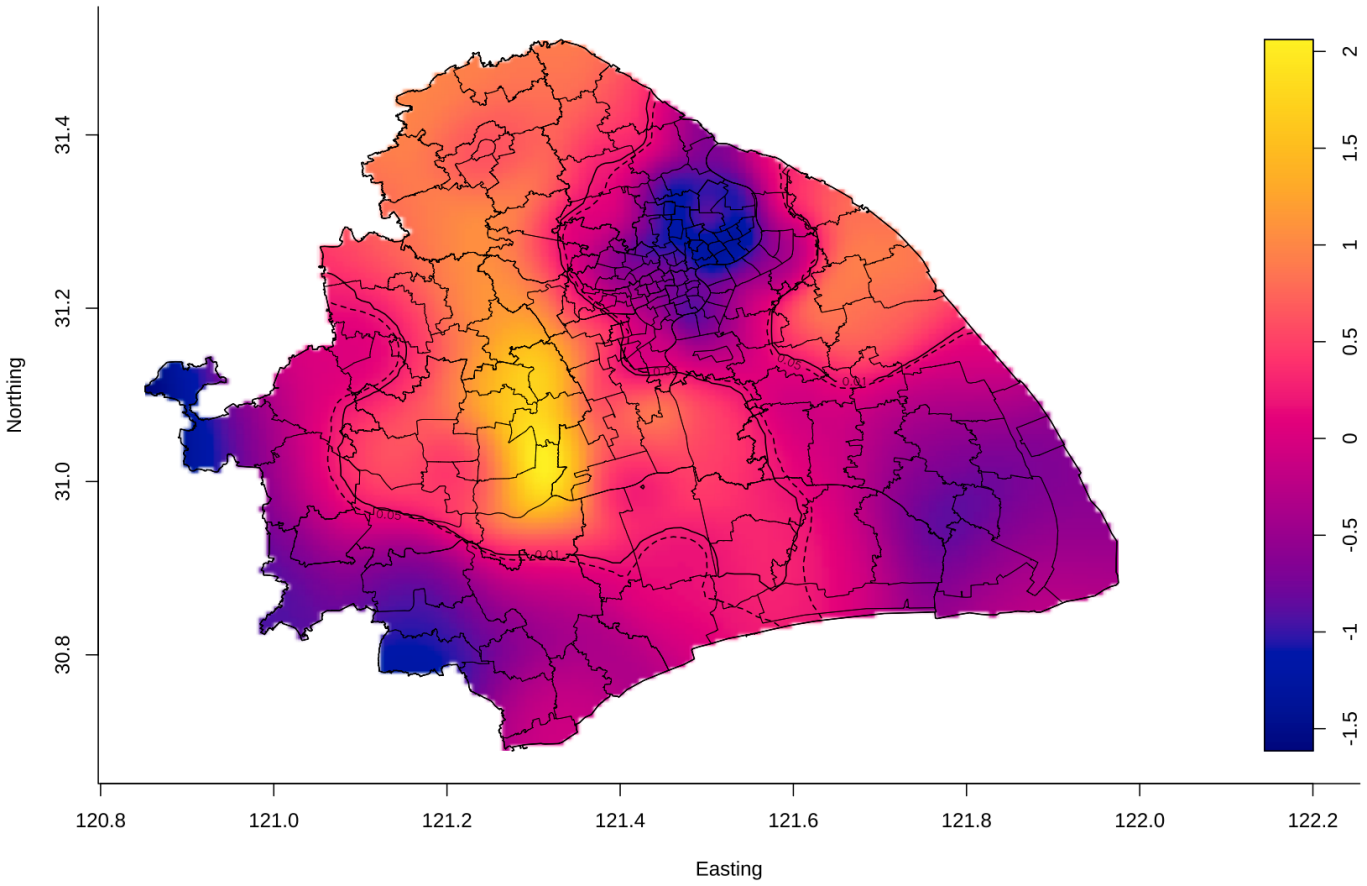
Adaptive bandwidth spatial log-relative risk surfaces of internal migrant TB. The gradient color scale represented the degree of the spatial log-relative risk. The outer contour indicated the *p*-value was 0.05, and the inner contour denoted the risk surface with a statistically significant (*p* = 0.01), in which the risk of internal migrant TB was relatively higher than that of the resident TB.

Despite the utilized area data, we conducted a spatial relative risk analysis of the geographic point data (Figure 4). We identified the relatively higher risk counties (Chedun, Zhongshan, and Xinqiao counties located on the border between Songjiang and Minhang Districts) of internal migrant TB than resident TB. Overall, we detected significant spatial heterogeneity of TB in Shanghai, and internal migrants seemed to play a role in it. It was important to explore the formation of spatial heterogeneity and the role of internal migrants in TB in Shanghai.

### Risk factors of spatial heterogeneity

#### Individual factors associated with the risk of TB

To explore possible risk factors of the spatial heterogeneity identified in Figure 3, we used univariable and multivariable logistic regression to evaluate the potential risk factors of spatial clusters. The multivariable logistic analysis result (Table 2) showed risk factors that were associated with having a high-clustering county included migrants (aOR, 1.85; 95%CI, 1.65–2.08), sputum AFB test negative (aOR, 1.55; 95%CI, 1.40–1.70), and active screening (aOR, 3.13; 95%CI, 2.60–3.77). Surprisingly, labour workers had 6.06 times the risk of being in a high-clustering county than commercial service staff.

**Table 2:** Multivariable logistic regression on the risk factors of TB spatial high clustering in Shanghai.

Characteristics	Low-clusters		Non-clusters		High-clusters		*p* value^a^	Multivariable regression^a^	
									*n* = 83	(%)	*n* = 25,071	(%)	*n* = 2,229	(%)
aOR (95%CI)	*p* value
Demographic attribute							<0.001		
Resident	51	(61.45)	14,909	(59.47)	774	(34.72)		Ref	
Migrant	32	(38.55)	10,162	(40.53)	1,455	(65.28)		1.85 (1.65, 2.08)	<0.001
Gender							0.103		
Female	27	(32.53)	7,266	(28.98)	683	(30.64)		..	
Male	56	(67.47)	17,805	(71.02)	1,546	(69.36)		..	
Age group, years							<0.001		
0–14	0	(0.00)	79	(0.32)	4	(0.18)		0.43 (0.15, 1.22)	0.113
15–24	11	(13.25)	4,166	(16.62)	616	(27.64)		1.06 (0.86, 1.31)	0.594
25–44	23	(27.71)	7,709	(30.75)	833	(37.37)		0.84 (0.69, 1.03)	0.090
45–64	29	(34.94)	7,612	(30.36)	457	(20.50)		0.73 (0.61, 0.87)	<0.001
≥65	20	(24.10)	5,505	(21.96)	319	(14.31)		Ref	
Occupation							<0.001		
Commercial service	3	(3.61)	1,141	(10.59)	41	(1.84)		Ref	
Labour worker	11	(13.25)	4,610	(18.39)	1,039	(46.61)		6.06 (4.39, 8.35)	<0.001
Farmer	0	(0.00)	1,435	(5.72)	237	(10.63)		6.61 (4.62, 9.46)	<0.001
Medical staff	0	(0.00)	120	(0.48)	5	(0.22)		1.43 (0.55, 3.71)	0.458
Student/teacher	3	(3.61)	937	(3.74)	121	(5.43)		3.62 (2.49, 5.27)	<0.001
Retirement	30	(36.14)	6,084	(24.27)	212	(9.51)		1.48 (1.02, 2.15)	0.038
Housework/unemployed	9	(10.84)	3,920	(15.64)	296	(13.28)		2.30 (1.65, 3.22)	<0.001
Other	27	(32.53)	6,824	(27.22)	278	(12.47)		1.30 (0.93, 1.82)	0.124
TB history							<0.001		
New case	73	(87.95)	21,562	(86.00)	1,998	(89.64)		..	
Retreated case	10	(12.05)	3,509	(14.00)	231	(10.36)		..	
Sputum AFB test							<0.001		
Positive	66	(79.52)	18,831	(75.11)	1,479	(66.35)		Ref	
Negative	17	(20.48)	6,134	(24.47)	747	(33.51)		1.55 (1.40, 1.70)	<0.001
Other	0	(0.00)	106	(0.42)	3	(0.13)		0.43 (0.13, 1.37)	0.152
Patient source							<0.001^b^		
Passive screening	82	(98.80)	24,534	(97.86)	2,040	(91.52)		Ref	
Active screening^c^	1	(1.20)	507	(2.02)	184	(8.25)		3.13 (2.60, 3.77)	<0.001
Other	0	(0.00)	30	(0.12)	5	(0.22)		1.49 (0.56, 3.98)	0.422
Diagnosis delay							0.127		
0–2 w	18	(21.69)	6,360	(25.37)	550	(24.68)		..	
2 w–1 m	30	(36.15)	7,465	(29.78)	703	(31.54)		..	
1–3 m	24	(28.92)	9,282	(37.02)	782	(35.08)		..	
3–6 m	6	(7.23)	1,450	(5.78)	148	(6.64)		..	
6 m–1 y	5	(6.02)	514	(2.05)	46	(2.06)		..	

aOR, adjusted odds ratio; CI, confidence interval; TB, tuberculosis; AFB, acid fast bacilli.

a Non-clusters vs high-clusters.

b Result of Fisher exact test.

c Include contactor investigation (0 cases in low-clusters, 12 cases in non-clusters, 4 cases in high-clusters).

We also performed logistic regression on the high-clustering counties in Jiading District identified in Figure 3B. We found that patients being students or teachers were associated with the spatial clusters in this district (Supplementary Table S2).

#### Spatial factors associated with the risk of TB

Geographic covariates in different spatial regions could impact the incidence and prevalence of TB disease. Based on the hierarchical Bayesian analysis results with the BYM model (Table 3; Supplementary Table S3), we found that industrial parks (RR, 1.420; 95%CI, 1.023–1.974) and migrants (RR, 1.121; 95%CI, 1.007–1.247) were the risk factors associated with the risk of incident TB in Shanghai. In contrast, the increasing *per capita* GDP (RR, 0.978; 95%CI, 0.960–0.995) in each county was associated with reduced TB incidence. We gained posterior risk, and residual relative risk plotted in Supplementary Figure S6 and found that the counties with posterior risk over 1 were in the counties with residual relative risk greater than 1. It suggested that these high-risk counties that might not be explained adequately by spatial factors might be caused by transmission.

**Table 3:** Hierarchical Bayesian modelling on the spatial risk factors of TB.

Parameter	Relative risk	95%CI
Presence of industrial parks	**1.420**	**(1.023, 1.974)**
Percentage of migrants (per 10% increase)	**1.121**	**(1.007, 1.247)**
Population density (per 1,000 increase)	1.011	(0.999, 1.024)
Per capita GDP (per 1,000 increase)	**0.978**	**(0.960, 0.995)**
Household size (per 1 increase)	2.108	(0.851, 5.419)
Number of rooms per household (per 1 increase)	0.834	(0.539, 1.284)

CI, confidence interval. The bold value indicates that the 95%CI did not include 1.

### Origins of internal migrant TB patients in Shanghai

**Figure 5:**
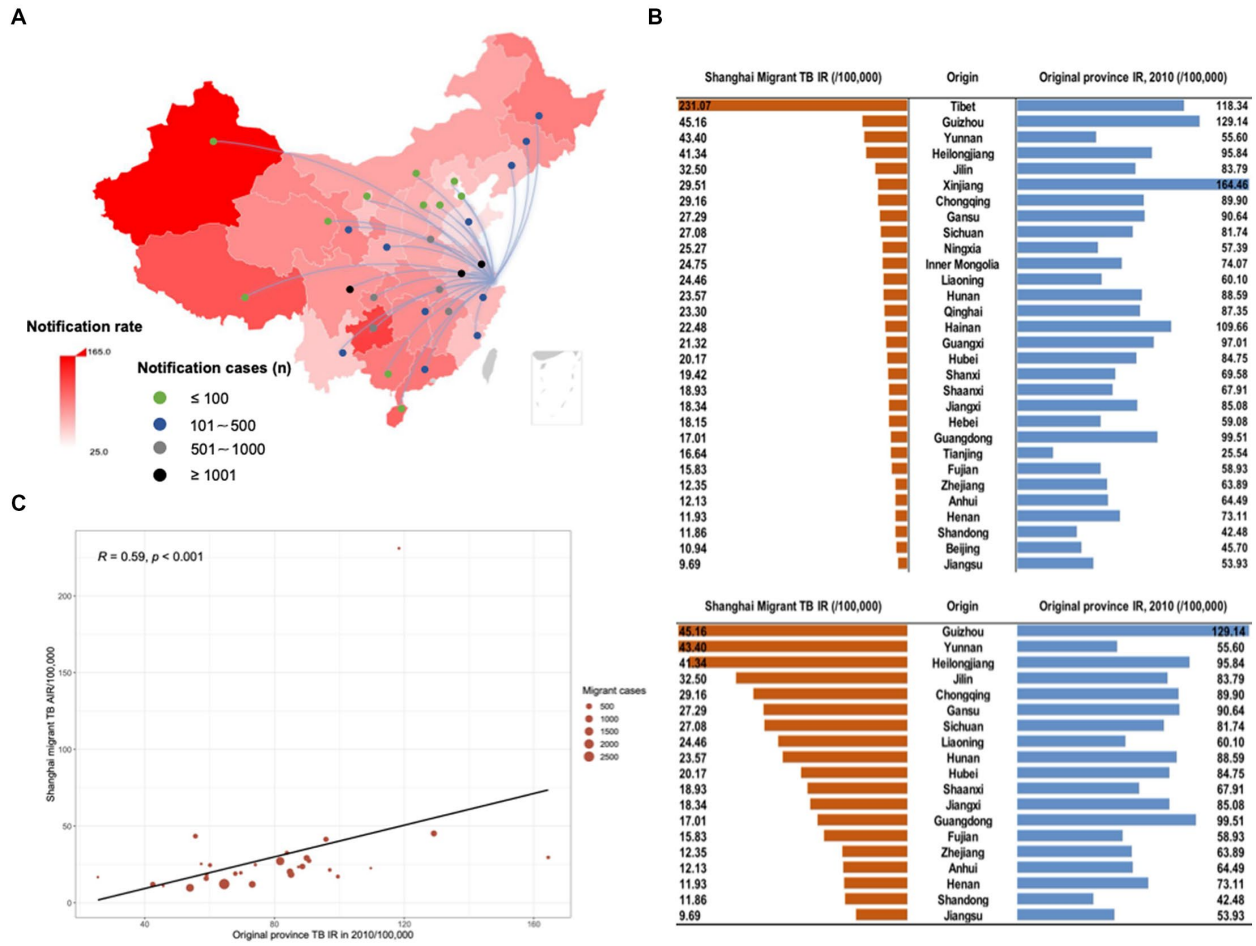
The origin of household registration among migrant TB cases in Shanghai. The background color of the map **(A)** indicated the notification rate of TB according to 2010 TB notification rate in each province in mainland China. Lines and dots presented the origin of migrant TB cases and the number of TB cases of the migrant population from the corresponding provinces moving to Shanghai, respectively. **(B)** The notification rate of TB between migrants who moved to Shanghai from each province and the original province. **(C)** Scatter plot of correlation analysis of TB notification rate reported in the original province and Shanghai migrant TB average annual notification rate. The size of the red circle in the figure indicated notified TB cases among extra-provincial internal migrants in Shanghai.

To understand the mechanisms driving TB risk among internal migrants, we explored the original place of household registration of internal migrant TB patients in Shanghai in Figure 5. Most internal migrant patients were originally from Anhui (21.71%), Sichuan (11.63%), and Jiangsu (10.02%) provinces (Figure 5A). The notification rate of TB between internal migrants who moved to Shanghai from each province and the original province had a positive correlation (*R* = 0.59, *p* < 0.001, Figures 5B,C). It suggested the original provinces’ TB background characteristics (e.g., TB incidence and burden) had a possible impact on the incident TB among the accordant internal migrants in Shanghai.

## Discussion

Our findings revealed the impact of population structure and migration on TB disease distribution during the urbanization in the past decades in eastern China. Significant spatial heterogeneity existed in the distribution of TB in Shanghai. The high clusters in Shanghai TB were mainly concordant with the high clusters of internal migrant TB. Population mobility was the main driving factor of the TB burden in the megacity in China, and the notification rate of TB among migrants was correlated with the original provincial TB incidence.

This study identified significant spatial heterogeneity of TB in Shanghai, especially among the internal migrant population. High clusters of internal migrant TB were mainly in certain counties in Songjiang, Minhang, and Jiading Districts, all designated industrial districts in Shanghai (26). In general, the personal life, work, and communication patterns of the migrant population were mainly in a clustered environment, which could promote the role of the migrant population in the development of TB disease and lead to a regional high TB burden (27). These social and residential patterns and the related spatial aggregation could be the reason for forming spatial clusters in the current study. The resident TB cases, especially the older adult, were spatially clustered in the downtown, which could also lead to clustering transmission (16). In addition, the previous research found that local high clusters related to local transmission (16). The clustering pattern identified for Songjiang District in our study using kernel density estimation (Supplementary Figures S7C,D) was similar to the previous reported. Thus, the high clusters in other districts in Shanghai identified in our study might be caused by local transmission, but this warrant further molecular analysis.

In previous study (16), migrants infected with TB had two patterns: pre-migration infection and transmission or recent infection after migration. Internal migrant TB cases in Shanghai might have been infected with *Mycobacterium tuberculosis* before coming to Shanghai and then developed symptoms in Shanghai. Like the epidemiology of TB among international immigrants, the internal migrant TB in our study also showed a significant correlation with the background notified incidence of TB in their places of origin (28, 29). Therefore, the TB background characteristics in the original residential area of migrants might be one of the drivers of TB burden in their destinated cities. Meanwhile, the routine occupational health examinations of migrant workers in high-endemic areas can be improved to actively find the TB cases. Aside from the reactivation of latent TB infection (LTBI) acquired before migration, the high spatial clusters among internal migrants could have also resulted from the transmission or recent infection after migration. A previous model study in one of the Shanghai districts estimated that approximately 43% of migrant cases would result from recent infection (18). Together these findings suggest that efficient interventions are needed to interrupt the transmission.

Meanwhile, the mobility of internal migrants between their work region and the center of the district (i.e., usually the medical, entertainment, and shopping centers) could lead to spill-out transmission across counties (16). This could also explain the finding we observed in Jiading District-another industrial district similar to Songjiang in Shanghai-in which high clusters in the district center were found in counties with many surrounding industrial parks. Such transmission events could be caused by casual contact with TB infectors (30).

We also noticed that active TB screening was significantly associated with forming high clusters. Most active screenings of TB were from internal migrants due to the forced occupational health examination, particularly in the large industrial parks (e.g., Songjiang and Minhang), which increased the possibility of TB case notification among migrants. The notification rate of TB was lower than the estimated level (31, 32), which was associated with failing to diagnose TB patients and report them to the national registry (33). Despite the contribution of TB cases from active case examination among internal migrant, it only accounts for 4.64% and 6.61% of the overall TB cases notified in Songjiang and Minhang districts, respectively (Supplementary Figure S8). The social and medical benefits of migrants were significantly diminished, and their mobility also made it more challenging to complete DOTS (Directly Observed Treatment, Short-Course). Thus, we should strengthen the ability to actively screen cases among migrants in high-clustering regions, which received less attention (34), and identify potential cases as early as possible to interrupt the transmission. Meanwhile, the active screening of cases among the residents cannot be ignored.

This study has several limitations. Firstly, we used the home address reported during the confirmation of TB patients, which may not necessarily be where the cases were infected or transmitted. Secondly, the retrospective study design may limit the power to examine the associated risk factors; a further investigation, such as a genomic epidemiology study, was warranted to explore the transmission of TB in the role of formed high-risk counties. Finally, due to the health insurance and TB stigma, we may miss some TB cases among migrants who could leave Shanghai after the diagnosis of TB; however, the healthcare resources in Shanghai are relatively rich compared to the other provinces where those migrants came from, this influence could be limited.

## Conclusion

In summary, our study elucidated that the migrant population played an essential role in both the spatial heterogeneity of TB and its burden in one of the largest megacities in China. Targeted interventions including active case finding in areas with high clusters of TB, primarily focusing on migrant populations, may be more effective in achieving TB control goals. Meanwhile, further model prediction and molecular epidemiology analysis are needed to identify spatial cluster risk factors to interrupt transmission accurately.

## Data availability statement

The original contributions presented in the study are included in the article/Supplementary material, further inquiries can be directed to the corresponding authors.

## Ethics statement

The study and the use of data were reviewed and approved by the Ethical Review Committee at the Shanghai Municipal Center for Disease Control and Prevention (2011630).

## Author contributions

HL, RZ, ZW, XS, and CY conceived and designed this study. HL, ZW, and XS participated in data management. HL, RZ, ML, and JW cleaned the data. HL, RZ, and CY performed the data analysis. HL and CY wrote preliminary drafts of the study and collected revisions from all authors. CY and XS had full access to all the data in the study and had final responsibility for the decision to submit for publication. All authors contributed to the article and approved the submitted version.

## Funding

The study was supported by the National Natural Science Foundation of China (Grant No. 81872679), Science and Technology Innovation Plan of Shanghai Science and Technology Commission (Grant No. 21DZ2202400), Shanghai Municipal Health Commission (20204Y0212), the Shenzhen Nanshan district San-Ming project (SZSM202103008), and the Pearl River Talent Plan Innovation and Entrepreneurship Team Project of Guangdong Province (2021ZT09Y544).

## Conflict of interest

The authors declare that the research was conducted in the absence of any commercial or financial relationships that could be construed as a potential conflict of interest.

## Publisher’s note

All claims expressed in this article are solely those of the authors and do not necessarily represent those of their affiliated organizations, or those of the publisher, the editors and the reviewers. Any product that may be evaluated in this article, or claim that may be made by its manufacturer, is not guaranteed or endorsed by the publisher.
