# Supplementary material for: Assessing the spatial heterogeneity of tuberculosis in a population with internal migration in China: a retrospective population-based study
Source: Front Public Health. 2023 May 30;11:1155146. doi: 10.3389/fpubh.2023.1155146 (PMC10266412; doi:10.3389/fpubh.2023.1155146)
Supplement: Supplementary file 1 [file Data_Sheet_1.docx]

Supplementary Material

Assessing the Spatial Heterogeneity of Tuberculosis in a Population with Internal Migration in China: A Retrospective Population-based Study

**Honghua Lin^1†^, Rui Zhang^1†^, Zheyuan Wu^2,3†^, Minjuan Li^1^, Jiamei Wu^1^, Xin Shen^2,3*^, Chongguang Yang^1,4,5*^**

*** Correspondence:**

Chongguang Yang: [yangchg9@mail.sysu.edu.cn](mailto:yangchg9@mail.sysu.edu.cn);

Xin Shen: shenxin@scdc.sh.cn

**List for supplementary material**

**Supplementary Table S1.** Getis-Ord General G statistics of TB in Shanghai, 2009-2016.

**Supplementary Table S2.** Multivariable Logistic Regression on the Risk Factors of Internal Migrant TB Spatial High Clustering in Jiading District.

**Supplementary Table S3.** Model Fitting Effect Comparison for Hierarchical Bayesian Models.

**Supplementary Figure S1.** Study data inclusion and study area.

**Supplementary Figure S2.** Distribution map of migrant population proportion and notification rate.

**Supplementary Figure S3.** Number of TB cases in migrant and resident population and notification rate in different years.

**Supplementary Figure S4.** The Age-Sex pyramid of TB among migrants and local residents.

**Supplementary Figure S5.** Counties with high clusters of overall and migrant tuberculosis in Shanghai, 2009-2016.

**Supplementary Figure S6.** The posterior risk and residual relative risk of TB with Hierarchical Bayesian model.

**Supplementary Figure S7.** Kernel density estimation of point data of migrant and resident TB.

## Supplementary Figure S8. Active screening in districts in Shanghai.

| **Supplementary Table S1. Getis-Ord General G statistics of TB in Shanghai, 2009-2016.** | | | |
| --- | --- | --- | --- |
| average annual notification rate | General G | Z-score | *p* value |
| Overall TB | 0.00522 | 2.611 | 0.009 |
| Migrant TB | 0.00539 | 4.213 | <0.001 |
| Resident TB | 0.00525 | 2.919 | 0.004 |

| Supplementary Table S2. Multivariable logistic regression on the risk factors of internal migrant TB spatial high clustering in Jiading District. | | | | | | | |
| --- | --- | --- | --- | --- | --- | --- | --- |
| Characteristics | Non-clusters  n=839 (%) | | High-clusters n=226 (%) | | *p* value | Multivariable regression | |
|  |  |  |  |  |  | *aOR* (95%*CI*) | *p* value |
| Gender |  |  |  |  | 0.475 |  |  |
| Female | 305 | (36.35) | 88 | (38.94) |  | ... |  |
| Male | 534 | (63.65) | 138 | (61.06) |  | ... |  |
| Age group, years |  |  |  |  | 0.312^a^ |  |  |
| 0-14 | 8 | (0.95) | 1 | (0.44) |  | 0.05 (0.005, 0.56) | 0.015 |
| 15-24 | 226 | (26.94) | 69 | (30.53) |  | 0.42 (0.17, 1.02) | 0.055 |
| 25-44 | 441 | (52.56) | 109 | (48.23) |  | 0.40 (0.17, 0.94) | 0.036 |
| 45-64 | 139 | (16.57) | 35 | (15.49) |  | 0.42 (0.17, 1.04) | 0.060 |
| ≥65 | 25 | (2.98) | 12 | (5.31) |  | Ref |  |
| Occupation |  |  |  |  | 0.004 |  |  |
| Commercial service | 39 | (4.65) | 5 | (2.21) |  | Ref |  |
| Labour worker | 463 | (55.19) | 142 | (62.83) |  | 2.34 (0.90, 6.05) | 0.081 |
| Farmer | 34 | (4.05) | 6 | (2.66) |  | 1.31 (0.36, 4.76) | 0.679 |
| Student/teacher | 23 | (2.74) | 16 | (7.08) |  | 7.56 (2.29, 24.97) | <0.001 |
| Retirement | 19 | (2.27) | 5 | (2.21) |  | 1.06 (0.23, 4.85) | 0.944 |
| Housework/unemployed | 130 | (15.50) | 29 | (12.83) |  | 1.63 (0.59, 4.52) | 0.348 |
| Other | 131 | (15.61) | 23 | (10.18) |  | 1.35 (0.48, 3.80) | 0.566 |
| TB history |  |  |  |  | 0.864 |  |  |
| New case | 773 | (92.13) | 209 | (92.48) |  | ... |  |
| Retreated case | 66 | (7.87) | 17 | (7.52) |  | ... |  |
| Sputum AFB test |  |  |  |  | 0.807^a^ |  |  |
| Positive | 661 | (78.78) | 177 | (78.32) |  | ... |  |
| Negative | 173 | (20.62) | 47 | (20.80) |  | ... |  |
| Other | 5 | (0.60) | 2 | (0.89) |  | ... |  |
| Patient source |  |  |  |  | 1^a^ |  |  |
| Passive screening | 837 | (9.76) | 226 | (100) |  | ... |  |
| Active screening^b^ | 2 | (0.24) | 0 | (0) |  | ... |  |
| Diagnosis delay |  |  |  |  | 0.728^a^ |  |  |
| 0-2w | 252 | (30.04) | 77 | (34.07) |  | ... |  |
| 2w-1m | 235 | (28.01) | 57 | (25.22) |  | ... |  |
| 1-3m | 292 | (34.80) | 74 | (32.74) |  | ... |  |
| 3-6m | 45 | (5.36) | 13 | (5.75) |  | ... |  |
| 6m-1y | 15 | (1.79) | 5 | (2.21) |  | ... |  |
| *aOR*, adjusted odds ratio; *CI*, confidence interval; TB, tuberculosis; AFB, acid fast bacilli.  ^a^Result of *Fisher* exact test.  ^b^only health examination cases. | | | | | | | |

| **Supplementary** **Table S3. Model fitting effect comparison for Hierarchical Bayesian models.** | | | |
| --- | --- | --- | --- |
| Model | DIC | WAIC | loglikelihood |
| Model 1 | 19285.81 | 20109.34 | -9635.96 |
| Model 2 | 1682.59 | 1626.48 | -647.38 |
| Model 3 | 1684.95 | 1631.84 | -648.74 |
| Model 4 | 1682.82 | 1628.53 | -648.07 |
| Model 5 | 1683.24 | 1629.61 | -647.97 |
| DIC, deviance information criterion; WAIC, widely applicable information criterion.  Model 1: no random effects (standard Poisson regression).  Model 2: observation-level independent random effects, accounts for overdispersion and appropriately inflates uncertainty measures.  Model 3: intrinsic conditional autoregressive (ICAR) random effect, accounts for spatial correlation between the regions.  Model 4: Leroux CAR random effects, balance spatial and independent variability.  Model 5: ICAR random effect and independent random effect (Besag, York, Mollie (BYM)), accounts for spatial correlation while avoiding over smoothing. | | | |

We used hierarchical Bayesian modeling to explore the impact of spatial factors on TB in Shanghai. We tried five models (Supplementary Table S3) and found that the difference in the model DIC, WAIC, and loglikelihood values in models 2-5 was small. We finally selected the BYM model, which accounted for spatial correlation avoiding over-smoothing.

Supplementary Figure S1. Study data inclusion and study area. (A) Sample inclusion and study flowchart. (B) Geographic location of the study areas (Shanghai exclude Chongming county).

Supplementary Figure S2. Distribution map of migrant population proportion and notification rate. The proportion of internal migrants among all population (A). The spatial distribution of the notification rate of overall TB (B) and migrant TB (C) by counties in Shanghai, 2009-2016.

Supplementary Figure S3. Number of TB cases in migrant and resident population and notification rate in different years. The bar graph represents the number of cases, and the line graph represents the notification rate. Orange and blue correspond to migrant TB and resident TB, respectively.

Supplementary Figure S4. The Age-Sex pyramid of TB among migrants and local residents. The left and right sides of the dotted line in the middle represent female and male cases with TB, respectively. Red indicates migrant TB cases, while green indicates local resident TB cases.


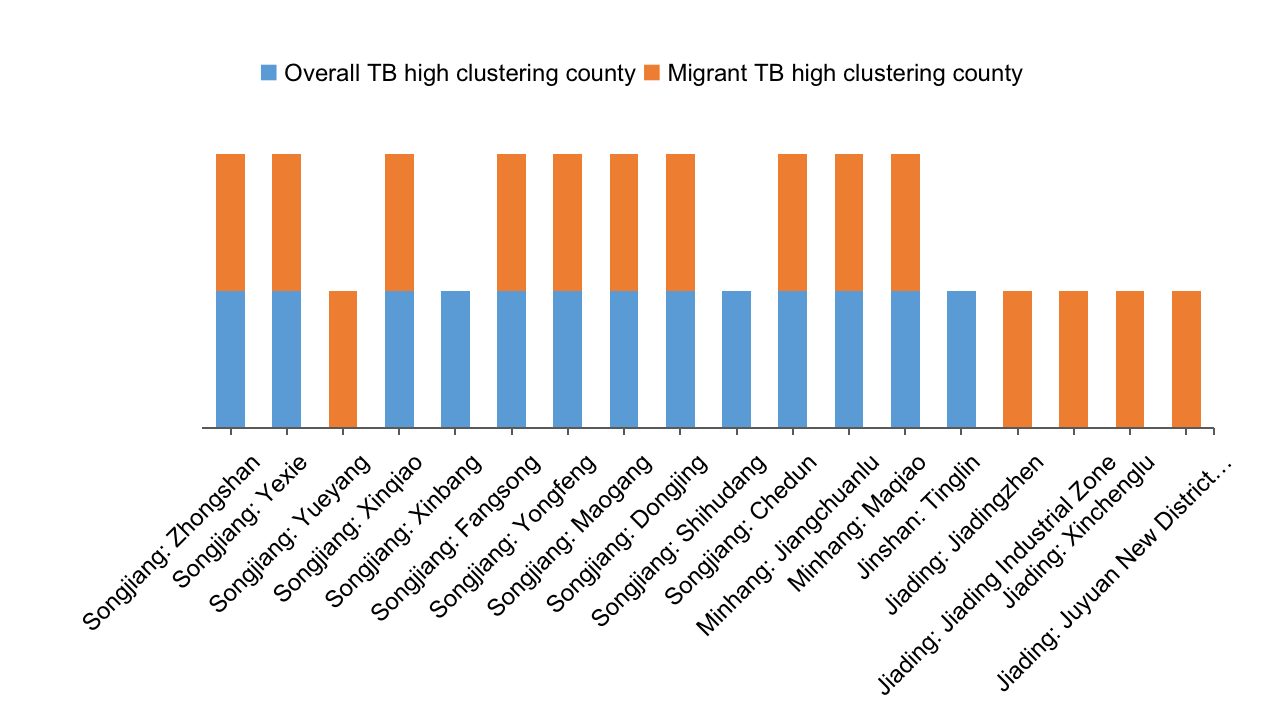


Supplementary Figure S5. Counties with high clusters of overall and migrant tuberculosis in Shanghai, 2009-2016. The blue and orange bars indicate overall TB high-clustering county and internal migrant TB high-clustering county, respectively. In our study, we identified 13 and 15 high-clustering counties with the hot spot analysis of the overall and migrant TB, respectively. Among them, 12 and 11 high-clustering counties were in Songjiang and Minhang Districts, respectively, and the high-clustering areas' overlapped rate of the overall TB and migrant TB reached 76.92% (10/13).

Supplementary Figure S6. The posterior risk and residual relative risk of TB with Hierarchical Bayesian model. (A) posterior risk; (B) residual relative risk.


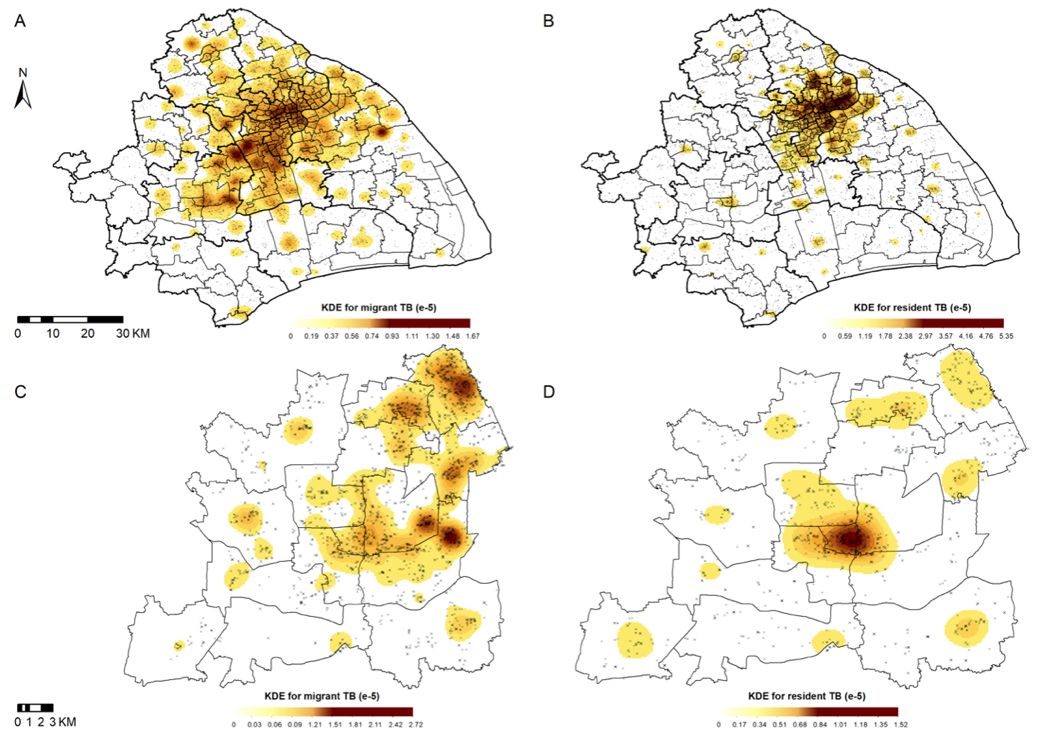


Supplementary Figure S7. Kernel density estimation of point data of migrant and resident TB. (A) Internal migrant TB in Shanghai, (B) resident TB in Shanghai, (C) internal migrant TB in Songjiang District, Shanghai, and (D) resident TB in Songjiang District, Shanghai.


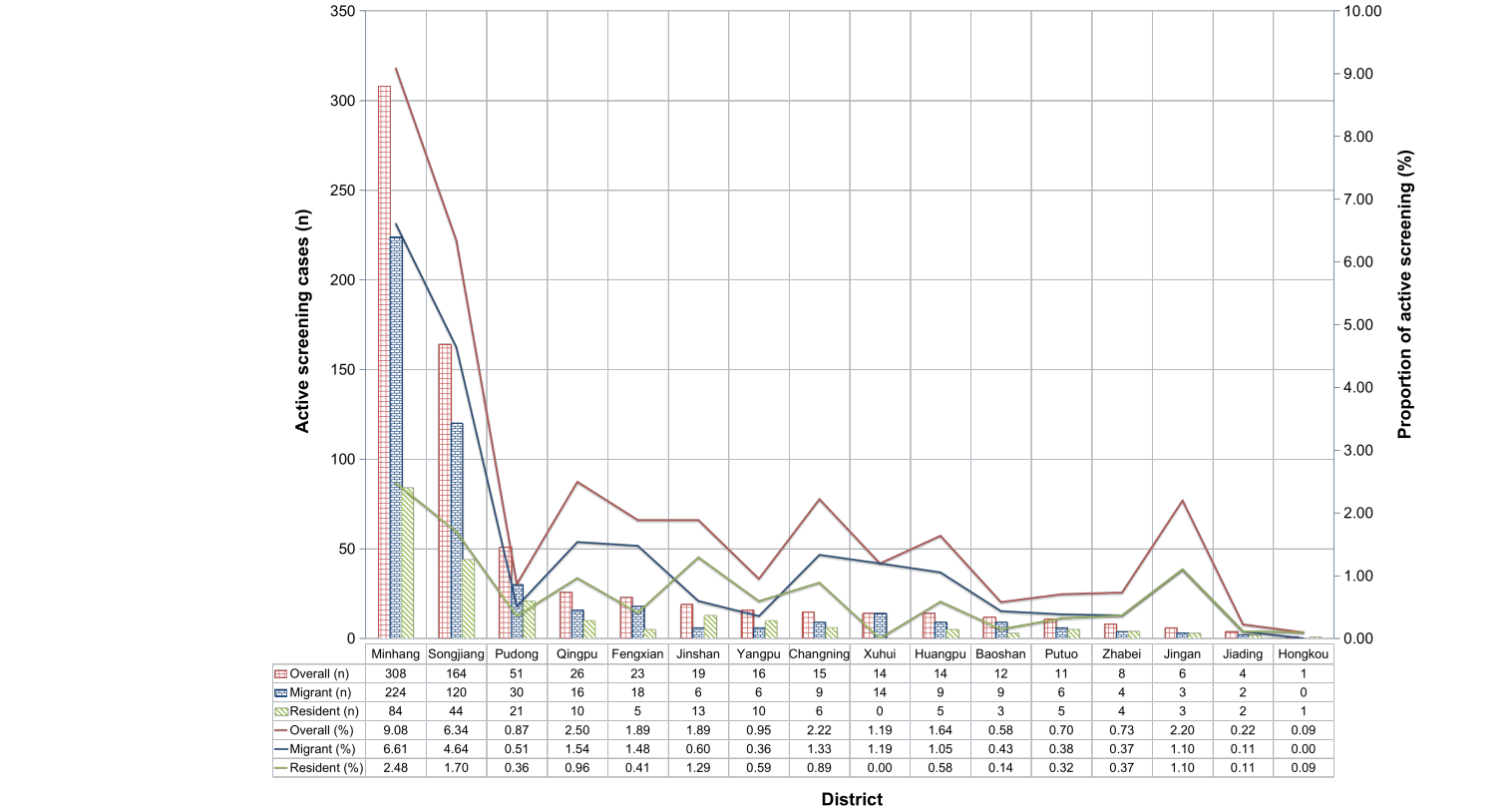


Supplementary Figure S8. Active screening in districts in Shanghai. The TB cases reported by active screening were mainly in Minhang and Songjiang Districts. The exact number of TB cases from active case finding was showed on the left y-axis. The proportion of TB cases from active screening among the total notified TB cases in each county was showed on the right y-axis.
